# Influences of Carrier Agents on Microbial Viability and Physicochemical Properties of Spray-Dried Coconut Yogurt

**DOI:** 10.3390/foods14223917

**Published:** 2025-11-17

**Authors:** Yanee Srimarut, Mattika Abhisingha, Nantanat Kosit, Jureeporn Dumnil, Preenapha Tepkasikul, Ausjima Poomkleang, Marisa Raita, Chetsadaporn Pitaksutheepong, Yuwares Malila

**Affiliations:** 1 Food Biotechnology Research Team, National Center for Genetic Engineering and Biotechnology, 113 Thailand Science Park, Pathum Thani 12120, Thailand; mattika.abh@biotec.or.th (M.A.); nantanat.kos@ncr.nstda.or.th (N.K.); jureeporn@biotec.or.th (J.D.); preenapha@biotec.or.th (P.T.); chetsadaporn@biotec.or.th (C.P.); 2 Enzyme Technology Research Team, National Center for Genetic Engineering and Biotechnology, 113 Thailand Science Park, Pathum Thani 12120, Thailand; ausjima.poo@ncr.nstda.or.th (A.P.); marisa.rai@biotec.or.th (M.R.); 3 Food Science and Technology Association of Thailand (FoSTAT), 50 Amon Bhumirat Bld., Bangkok 10903, Thailand; yuwaresm@gmail.com

**Keywords:** coconut yogurt, non-dairy alternative, plant-based alternative, spray drying

## Abstract

Plant-based fermented coconut yogurt, valued for its functional properties, requires transformation into a shelf-stable powder, necessitating carriers to overcome particle stickiness and preserve probiotic viability. The objective of this study was to investigate the influence of polysaccharide carriers (maltodextrins DE 2, 10, and 19, and resistant dextrin) on processing efficiency, physicochemical stability, and lactic acid bacteria (LAB) viability. The feed, standardized to 15% total solids (initial LAB counts of 8.54 log CFU/g), was spray-dried at a 120 °C inlet temperature and a 65 °C outlet temperature. The drying condition reduced LAB viability by two log cycles regardless of the tested carriers. Maltodextrin DE 19 showed the highest powder yield, the lowest water activity, and a higher water solubility index. No significant differences in bulk density, pH, titratable acidity, and lactic acid content were observed among samples. Low-DE maltodextrins (DE 2 and 10) demonstrated significantly higher retention of sensitive malic and citric acids compared to DE 19. The current findings suggested that high-DE carriers provided beneficial effects on physical processing via kinetic shell formation, while low-DE carriers were able to protect against the loss of small organic acids. Overall, the study lays a foundation for spray-dried carrier development for coconut yogurt.

## 1. Introduction

Plant-based yogurt alternatives have gained popularity as the products address consumer demand for dairy-free, allergen-free, and health-promoting aspects [1]. Among plant-based alternatives, commercial coconut yogurt products have achieved sensory profiles, including appearance, aroma, and flavor, as well as consumer acceptance comparable to traditional dairy yogurt [2]. Additional advantages of fermented coconut yogurt lie in its potential health benefits. Coconut is rich in medium-chain fatty acids [3] and is a good source of minerals [4]. Fermented coconut yogurt can be produced by blending coconut water with young coconut meat and fermenting it with lactic acid bacteria (LAB) similar to those used in dairy yogurt. These LABs (e.g., Lactobacillus and Streptococcus species) actively ferment the coconut sugars, producing organic acids (mainly lactic acid, with minor acetic acid in heterofermentative strains). With the plant-based dairy alternatives sector growing 11% annually in Europe [1], coconut yogurt represents a valuable innovation for the health food market. However, the product has a short shelf life and requires a cold chain for storage and distribution. Therefore, it is of interest to transform the coconut yogurt into a shelf-stable dried powder.

Spray drying is a cost-effective and scalable technique for food powders. By passing liquid food into a hot air stream, water can be rapidly evaporated to yield a powdered product with low moisture (often <5% by weight) [5]. Such low moisture content contributes to long-term stability and, therefore, a prolonged shelf life. Still, this technique poses some challenges when applied to complex foods that are rich in sugars and organic acids. In general, moisture is evaporated from the foods during drying, leaving other components solidified into powder [6,7]. The temperature of the powder must be lower than its glass transition temperature (Tg) in order to stay in the glassy state. However, sugars (e.g., glucose, lactose, galactose, and fructose) and organic acids (e.g., lactic acids, ascorbic acids, and citric acids) exhibit low Tg [6,7] and high hygroscopicity [6]. As a consequence, the spray-dried food particles remain in a rubbery state and can interact with other molecules, including the dry surface of the spray dryer. This phenomenon leads to stickiness problems and low yield of the spray-dried powder [7,8]. Another challenge in drying yogurt is to preserve the viability of beneficial microbes. Conventional spray drying involves inlet air temperatures of 120 °C to 180 °C [9,10]. Without an optimal protective technique, LAB populations are often significantly reduced by more than 1–3 log CFU during spray drying [9].

To overcome these issues, solid carriers and emulsifiers are generally included in the food before being subjected to the spray dryer. Maltodextrin, a common spray-drying carrier, can raise the overall Tg of the mixture [6]. In addition, the carriers can increase the solid content and form a structural layer around sensitive components, resulting in a free-flowing powder [6]. One factor affecting such properties is the molecular structure of those polysaccharides. The larger structure of maltodextrin, i.e., the lower dextrose equivalent (DE) value, tends to have higher Tg. Silalai and Roos [11] analyzed Tg of milk solids with maltodextrins with different DEs and observed that Tg was higher for milk solids containing DE9 maltodextrin than those containing DE17. Similarly, a droplet-drying study reported that low-DE maltodextrins exhibit higher Tg than high-DE maltodextrins (DE ≥ 12) under equivalent conditions [12]. Apart from maltodextrin, an application of resistant dextrin (RD), a water-soluble starch-derived short-chain polysaccharide, as a spray-drying carrier is of interest. RD is produced from the hydrolytic debranching process of α-1,4- and 1,6-glycosidic bonds in starch molecules [13]. During the dextrinization, the branched structure is formed with new bonds, including α-1,2-, α-1,3-, α-1,6-, β-1,6-, and β-1,2-bonds [14]. Not only does the structure of RD allow thermal stability, but it also has anti-digestive properties with less impact on the taste of food products [13].

While general principles from dairy yogurt or other model systems exist, the studies describing the effects of either maltodextrin or RD as a carrier for coconut yogurt are still limited. Based only on the available literature, it is hypothesized that the low-DE maltodextrin can provide better protective effects and better yield than those with a high DE value. Therefore, the objectives of the current study were to investigate the effects of maltodextrin with different DE values (i.e., DE 2, DE 10, and DE 19) and resistant dextrin on physical characteristics, microbial viability, and organic acids of spray-dried coconut yogurt.

## 2. Materials and Methods

### 2.1. Materials and Sample Preparation

Young coconuts were purchased from a local store (Damnoen Saduak, Ratchaburi, Thailand). Fermented coconut yogurt was prepared by blending coconut meat and coconut water at a ratio of 1:1. The starting cultures, containing lactic acid bacteria (LAB), were inoculated into the pasteurized coconut mixture. The mixture was incubated at 40 °C for 6 h until the pH value of the mixture reached 4.3. Proximate composition and LAB count of the fermented coconut yogurt are shown in Table 1. Maltodextrin (DE10-12) was purchased from Ma-Jusmin Group (Bangkok, Thailand), DE19 from BPNovitat (Bangkok, Thailand), and DE2 and resistant dextrin (RD) from Chanjao Longevity Co., Ltd. (Bangkok, Thailand).

### 2.2. Spray Drying

The fermented coconut yogurt was mixed with either maltodextrin or RD to reach a solid content of 15%. Spray drying was conducted using a laboratory-scale Büchi B-290 Mini Spray Dryer (Büchi Labortechnik AG, Flawil, Switzerland). The mixed solution of coconut yogurt was kept under agitation throughout the spray-drying process using a magnetic stirrer. The spray dryer was operated at an inlet air temperature of 120 °C, a main air flow aspirator rate of 100% (38 m^3^/h), an atomized air flow rotameter setting of 40 mm in height (473 L/h), and a pump rate for liquid of 4.5–6.0 mL/min. The settings resulted in an outlet temperature of approximately 65 °C. Dried powder was collected from both the collection vessel and the cyclone wall.

Yield of the spray-dried powder was calculated as the mass of the weight of the resulting powder collected after the spray drying divided by the mass of total solid content in the feed. The yield was expressed as a percentage (see Equation (1)).

(1) $$Yield\;\left(\%\right)=\frac{Weight\;of\;collected\;powder}{Total\;weight\;of\;solids\;in\;feed}\times 100$$

### 2.3. Bulk Density

Bulk density of the powders was determined based on the method of Amidon et al. [15]. The spray-dried powder was poured into a graduated cylinder without tapping. The result was then expressed as the weight of the powder per volume as calculated using Equation (2). The measurement was carried out in triplicate.

(2) $$Bulk\;density\;\left(g/ml\right)=\frac{M}{V_{0}}$$

Here, *M* = mass in grams and *V*_0_ = untapped apparent volume in milliliters.

### 2.4. Moisture Content, Solid Content, and Water Activity

Moisture of the samples was determined using a moisture analyzer (Excellence Plus HX204, Mettler-Toledo, Inc., Greifensee, Switzerland). Solid content was then calculated by subtracting the amount of moisture from 100%. Water activity (a_w_) of the dried powder was analyzed using an electronic dew point water activity meter measuring system (AquaLab Series 3 TE, Decagon Devices, Inc., Pullman, WA, USA). The measurement of a_w_ was carried out at 25 °C. The measurements of moisture and a_w_ were performed in triplicate.

### 2.5. Microbial Cell Viability

To determine the LAB survival rate, the powder samples were rehydrated using sterile distilled water at a ratio of 1:9 by weight. For the enumeration of lactic acid bacteria, MRS with calcium carbonate agar plates was incubated anaerobically at 37 °C for 72 h. The average counts of 30–300 colonies were considered an acceptable detection range. The measurement of microbial cell viability was carried out in triplicate. The results were expressed in log CFU/g sample.

### 2.6. Solubility Index

The solubility index of the dried powder sample was analyzed following a modified method from Arya and Kumar [16]. In brief, 0.1 g of powder was mixed with 5 mL of distilled water. The mixture was vortexed for 30 s and then centrifuged at 3000× *g* for 10 min. The total solid of the supernatant was determined using a moisture analyzer (Excellence Plus HX204, Mettler-Toledo, Inc., Greifensee, Switzerland). The solubility index was performed in triplicate and expressed as a percentage for the ratio of solid content in the supernatant and the original sample weight.

### 2.7. pH and Titratable Acidity

The pH of the coconut yogurt and reconstituted powder sample was measured using a pH meter (Mettler-Toledo SevenEasy, Mettler-Toledo, Inc., Greifensee, Switzerland). As for titratable acidity, the dried samples were reconstituted in distilled water (10% *w*/*w*), followed by a titration with 0.1 N NaOH. Phenolphthalein was added as an endpoint indicator. Titratable acidity was measured in triplicate and expressed equivalently to lactic acid.

### 2.8. Organic Acids

The organic acid extraction from coconut yogurt powder was modified from Asarat et al. [17]. First, 4 mL of hexane was added to 200 mg of the sample and vigorously shaken to remove any lipids. Hexane was then removed from the sample via centrifugation (Eppendorf AG, Hamburg, Germany) at 10,000 rpm for 15 min at 4 °C, and the residual hexane was completely removed under a nitrogen stream. Subsequently, organic acids in the sample were extracted with 1.5 mL of 0.05 N perchloric acid using a vortex mixer at a speed of 2500 rpm for 5 min. After the sample was centrifuged at 10,000 rpm for 15 min at 4 °C, the supernatant was collected and further analyzed using Ultra-Performance Liquid Chromatography (UPLC).

The analysis of organic acids in coconut yogurt powder was performed in triplicate, following the method of Gardana et al. [18] using an Acquity HSS T3 column (2.1 mm × 150 mm, 1.8 μm) connected to a UPLC H-Class machine (Waters Corporation, Milford, MA, USA) with a column temperature of 40 °C. Various types of organic acids were eluted using two mobile phases: 0.1% formic acid (A) and acetonitrile/methanol at a 1:1 ratio (B), following the gradient program shown in Table 2 at a flow rate of 0.2 mL/min. UV absorbance was measured at a wavelength of 210 nm using a photodiode array (PDA). Quantification of organic acids was carried out by the external standard method. Five known concentrations (0.02–0.10 mg/g for acetic acid and citric acid or 0.10–2.00 mg/g for malic acid and lactic acid) of organic acids were analyzed, in triplicate, under similar conditions. The calibration curves demonstrating linearity with R^2^ > 0.99 were generated and used for calculating organic acid content, in the unit of mg per g powder sample, in the spray-dried samples.

### 2.9. Principal Component Analysis (PCA)

PCA was performed using Python 3.11 software following the method of covariance-based PCA on standardized data. A biplot was constructed based on the first two principal components (PCs). PC1 primarily represented compositional attributes (LAB, pH, acidity, and moisture), while PC2 captured functional properties (solubility and yield). To explore sample grouping, K-means clustering (k = 3) was applied to PCA scores to identify clusters. Cluster boundaries were visualized using convex hulls on the PCA biplot.

### 2.10. Statistical Analysis

To assess the effects of polysaccharide carriers, a one-way analysis of variance (ANOVA) was performed. The mean difference was determined following Duncan’s multiple range test. Statistical significance was set at *p* < 0.05.

## 3. Results

### 3.1. Yield and Bulk Density

**Figure 1:**
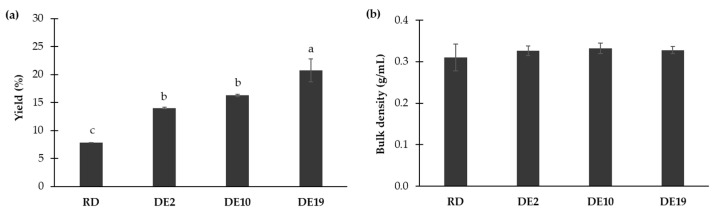
Effects of different polysaccharide carriers on (**a**) yield and (**b**) bulk density of spray-dried coconut yogurt. Bars and error bars represent means and standard deviations. Different letters above bars indicate significant differences (*p* < 0.05).

The effects of different carriers on the yield and bulk density of the spray-dried coconut yogurt are shown in Figure 1. The samples containing RD showed the lowest yield (*p* < 0.05), whereas the sample containing DE 19 showed the highest yield (*p* < 0.05). The samples added with maltodextrin DE 2 and DE 10 exhibited intermediate yields with no significant differences between each other (*p* ≥ 0.05). No significant differences in bulk density were observed among different maltodextrins (*p* ≥ 0.05). These findings suggest the beneficial impacts of maltodextrin with high DE values as a carrier for yield improvement of spray-dried coconut yogurt.

### 3.2. Moisture Content, Solid Content, and Water Activity

Table 3 shows the moisture, solid content, and a_w_ of the spray-dried coconut yogurt. The samples added with RD showed a lower moisture content than the samples containing DE 2 and DE 10. Among the samples containing maltodextrin as a carrier, moisture content tended to decrease as DE values increased (*p* < 0.05). Whilst the values of samples with DE 10 did not differ from those of DE 2 or of DE 19 (*p* ≥ 0.05). The results of solid content were in the opposite direction from those of moisture content. Focusing on a_w_, however, the samples with DE 19 showed lower a_w_ than the others (*p* < 0.05).

### 3.3. Microbial Cell Viability

**Figure 2:**
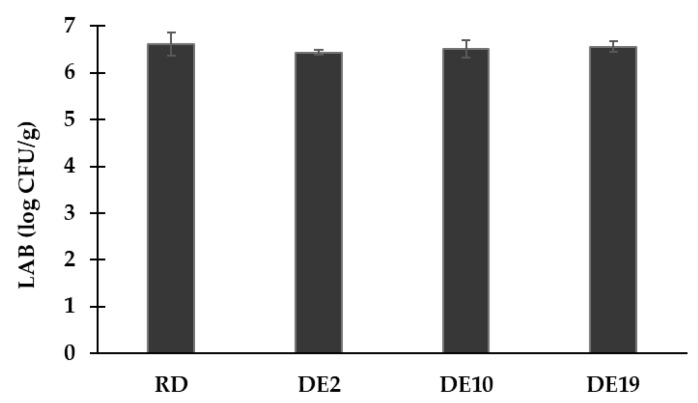
Microbial viability of spray-dried coconut yogurt as impacted by different polysaccharide carriers. Bars and error bars represent means and standard deviations.

The survival rates of lactic acid bacteria in the spray-dried powder samples were analyzed and are illustrated in Figure 2. The initial cell counts in the coconut yogurt were 8.54 log CFU/g; the viability of LABs in the spray-dried coconut yogurt varies from 6.43 to 6.61 log CFU/g. There was a 2-log reduction in LABs, indicating approximately 1% viability. The current results showed that different polysaccharide carriers did not exert a significant impact on the survival of lactic acid bacteria (*p* ≥ 0.05). The findings suggest the comparable protective effects of the tested carreirs on the survival rate of the beneficial microbes.

### 3.4. Solubility Index

**Figure 3:**
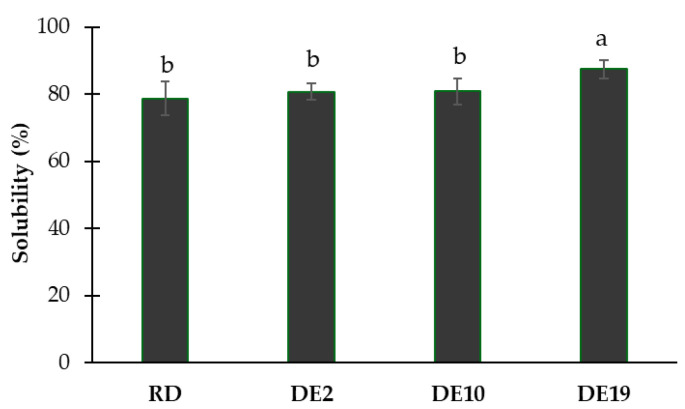
Solubility index of spray-dried coconut yogurt as affected by different polysaccharide carriers. Bars and error bars represent means and standard deviations. Different letters above bars indicate significant differences (*p* < 0.05).

The effects of different polysaccharide structures on the solubility of the spray-dried coconut yogurt are illustrated in Figure 3. The sample with maltodextrin DE 19 as a carrier exhibited the highest solubility (*p* < 0.05). The other samples showed no significant difference in solubility among each other (*p* ≥ 0.05). These findings suggest that the coconut yogurt powder containing maltodextrin DE 19 is dissolved in water to a greater extent compared to the other samples.

### 3.5. pH and Titratable Acidity

Table 4 shows the effects of different polysaccharide carriers on the pH and titratable acidity of the spray-dried coconut yogurt. There were no significant differences in pH and titratable acidity among the treatments (*p* ≥ 0.05). These results suggest that the different carriers did not exert a significant effect on the acidity of the spray-dried coconut yogurt when the samples were resuspended in water.

### 3.6. Organic Acids

**Figure 4:**
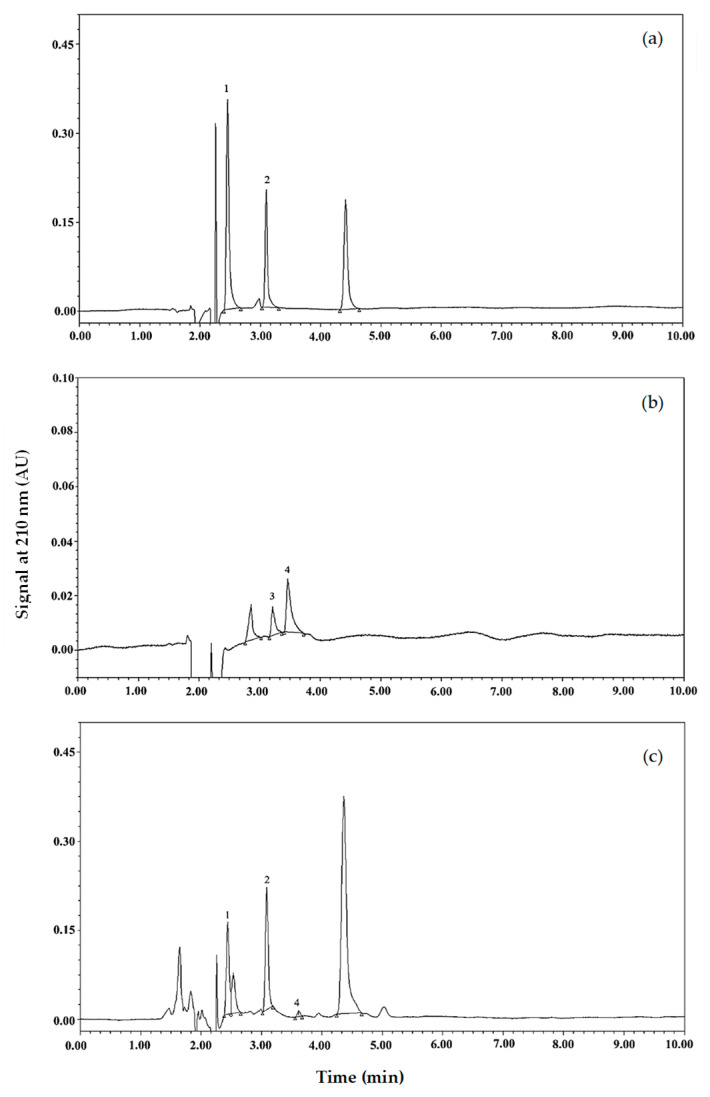
UPLC analysis of organic acids. (**a**,**b**) Chromatogram of organic acid standards. (**c**) Chromatogram of spray-dried coconut yogurt. Peaks 1, 2, 3, and 4 are L-malic acid, lactic acid, acetic acid, and citric acid.

In this study, four organic acids, including L-malic acid, lactic acid, acetic acid, and citric acid, were the primary focus. This is based on a previous report indicating oxalic, malic, ketoglutaric, acetic, citric, and succinic acids as naturally organic acids found in coconut water [19]. The presence of lactic acid was expected, as it is the major metabolic product of lactic acid bacteria used as yogurt culture. Chromatograms of organic acid standards and the samples are illustrated in Figure 4. L-lactic, malic, and citric acid were observed in the spray-dried powder (Figure 4c). The peak of acetic acid was, however, not detected. The quantity of each organic acid detected in the spray-dried samples is shown in Table 5. Citric acid and malic acid in the samples with maltodextrin DE 2 were not different from those of DE 10 (*p* ≥ 0.05), and the acids of those samples were higher than those of DE 19 and RD (*p* < 0.05). The RD-containing samples contained the lowest citric and malic acids among the samples (*p* < 0.05). On the other hand, no significant differences in lactic acid were observed among the samples (*p* ≥ 0.05).

### 3.7. Principal Component Analysis

**Figure 5:**
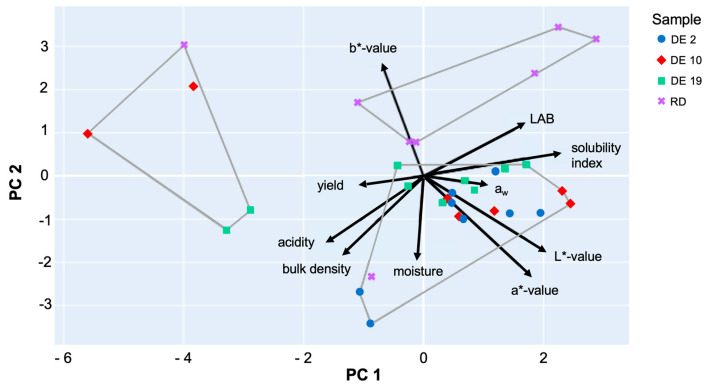
A Principal Component Analysis (PCA) biplot of the physicochemical and functional properties of the samples (DE2, DE10, DE19, and RD). The plot shows sample distribution along the first two principal components (PC1 and PC2), which, together, explain the majority of variance in the dataset. Black arrows indicate variable loadings, illustrating the contribution and direction of each original variable to the principal components.

PCA (Figure 5) reduced the multidimensional dataset into two principal components (PCs) explaining the majority of variance. K-means clustering (k = 3) applied to PCA scores identified three distinct clusters, corroborated by hierarchical clustering dendrograms. Cluster 1 was mainly composed of DE2 samples, characterized by high solubility (78%) and moderate yield (14%), with lower bulk density, suggesting a porous maltodextrin structure conducive to rapid rehydration. Cluster 2 included DE10 samples, showing moderate solubility (77%) and yield (15%), with slightly higher moisture content, indicating potential variability in drying efficiency. Cluster 3 was dominated by RD samples and some DE19, exhibiting lower solubility (63%) but the highest yield (18%) and higher bulk density, indicating a more compact structure that may hinder dissolution. This analysis demonstrates that carrier structure and process conditions significantly influence functional properties. High solubility correlates with lower bulk density and moderate yield, suggesting that optimizing drying parameters or carrier composition could enhance instant rehydration without compromising process efficiency.

## 4. Discussion

Two challenges of transforming coconut yogurt into a spray-dried powder form include overcoming the stickiness associated with high sugar and organic acid contents and ensuring the retention of functional LAB viability [6,20]. The main objective of this study was to investigate the structural role of polysaccharide carriers, specifically the dextrose equivalent (DE) of maltodextrin, in influencing the outcome of the spray-drying process. Overall, the results lay a foundation for formulating suitable carriers for coconut yogurt powder.

Focusing on the carriers, the performance of carriers in mitigating stickiness is generally evaluated based on their ability to elevate Tg of the overall mixture [6,7,21]. Such a property can ensure that the particles remain in a stable, non-sticky glassy state below the processing outlet temperature. For the stability of highly acidic and sugar-rich materials during spray drying, the selection of a carrier is also conventionally determined based on its Tg. The high-molecular-weight polymers (i.e., low-DE maltodextrins) were expected to raise the bulk Tg most effectively, thereby minimizing stickiness and maximizing yield [6,7,21]. The present findings were, however, contradicted by previous studies. Our results indicated that, for the spray-dried coconut yogurt powder, maltodextrin DE 19 provided better beneficial effects on yield, solid content, and a_w_ than the other carrier materials. The main aspects of such a contradiction could be due to the complexity of the food matrix. While food samples (i.e., fruit juices [5,8,22] and dairy yogurt [23]) in previous studies were mainly composed of acids, sugars, and, perhaps, beneficial microbes, the coconut yogurt also contained high fat and oligosaccharides (Table 1). Such components might also induce the glass transition phenomena during spray drying.

In addition, under the inlet temperature (120 °C), the process might be dominated by the mechanism of surface shell formation rather than bulk Tg properties [24]. With shorter polysaccharide chains, maltodextrin DE 19 would exhibit molecular mobility and accelerated diffusion rates to a greater extent compared to those of the low-DE maltodextrin and RD [25]. This kinetic advantage might allow the DE 19 carrier to rapidly migrate to the droplet surface and form a dense glassy shell before particle adhesion occurs on the dryer wall, thus maximizing the yield. On the other hand, a highly branched structure of the RD might physically hinder the necessary uniform and rapid surface migration required for effective anti-stickiness protection under the rapid kinetic conditions. In addition to the yield, the DE 19 formulation produced the most stable powder, characterized by the lowest water activity (a_w_ = 0.24) and superior solubility (Figure 3). This outcome further supported the idea that the rapidly formed, highly efficient shell provided by the high-DE carrier not only optimized process flow but also created a barrier for water retention.

Preservation of LAB viability in the spray-dried coconut yogurt was another challenge of product development. The initial LAB count of 8.54 log CFU/g (Table 1) was reduced by a moderate 2.0 to 2.4 log cycles (i.e., a 99% reduction) across all formulations, resulting in viable counts consistently above 6 log CFU/g (Figure 2). No statistical difference in LAB viability was observed among DE 2, DE 10, DE 19, or RD. The findings agreed well with a previous study in which spray drying led to reductions of 97 and >99% for *Streptococcus thermophilus* and *Lactobacillus delbrueckii*, respectively, in plain yogurt [26].

The preservation of organic acids is essential for maintaining the sensory characteristics of the fermented product. Organic acids could be volatile and lost during spray drying [27]. While lactic acid, the primary fermentation product, was retained uniformly across all samples, significant differences were observed in the retention of citric and malic acids. The findings suggested that the longer polymer chains of low-DE maltodextrin might provide a greater density of hydroxyl groups, promoting esterification and crosslinking with the polycarboxylic acids (i.e., citric and malic acids) under thermal exposure [7,28]. This chemical immobilization might prevent volatilization or degradation of those acids during the drying process. The failure of RD to retain citric acid suggested that its specific branched structure might not favor this polycondensation reaction under the process conditions.

Overall, the results demonstrated the roles of different structures of polysaccharide carriers during the spray drying of coconut yogurt. The findings pinpointed the crucial foundation for developing carriers for spray-dried food products containing a complex food matrix. For some products, such as coconut yogurt, the behavior of the carriers may not be simply explained by standard glass transition theories. For commercial applications, the choice of carrier must balance economic yield with chemical quality and the complex flavor profile of the coconut yogurt. However, limitations of the current study are worth noting. An empirical characterization of the powder’s physical state or microstructure is absent. Long-term storage viability of the LABs and the retention of sensitive flavor compounds over an extended shelf life (e.g., 6–12 months) remain to be explored. Moisture sorption studies and accelerated stability testing are essential to formally confirm the superior barrier function of the DE 19 matrix during ambient storage.

## 5. Conclusions

In summary, maltodextrin DE 19 exhibited better properties, in terms of yield, water activity, and resuspension, than the other tested carriers. However, the high-DE maltodextrins showed less retention of citric and malic acids of the coconut yogurt powder, which might affect the sensory characteristics of the powder. All maltodextrin carriers provided no differences in LAB viability, with viable counts remaining above the functional threshold of 6 log CFU/g. While highly functional, the current work is limited by the absence of long-term storage validation and particle morphological data. Future investigations underlining an in-depth analysis, including thermodynamic characterization along with the tailored blended carrier systems, are underway to simultaneously maximize the yield and fully preserve the overall spectrum of functional components in the product.

## Figures and Tables

**Table 1:** Proximate composition and lactic acid bacteria count in the fermented coconut yogurt.

Properties	Value
Moisture (%)	83.75 ± 0.07
Protein (%)	1.20 ± 0.00
Total fat (%)	6.22 ± 0.04
Carbohydrate (%)	8.17 ± 0.12
Ash (%)	0.67 ± 0.01
Lactic acid bacteria (log CFU/g)	8.54 ± 0.17

**Table 2:** UPLC gradient and mobile phase compositions used for organic acid analysis.

Time (min)	Mobile Phase A (Water with 0.1% Formic Acid)	Mobile Phase B (50/50 Methanol/Acetonitrile)
0.00	98	2
10.00	98	2
15.00	85	15
20.00	85	15
20.10	98	2
25.00	98	2

**Table 3:** Effects of different polysaccharide carriers on moisture, solid content, and water activity of spray-dried coconut yogurt.

Maltodextrin	Moisture Content (%)	Solid Content (%)	Water Activity
RD	3.91 ± 0.32 ^c^	96.10 ± 0.32 ^a^	0.265 ± 0.002 ^a^
DE 2	5.34 ± 0.44 ^a^	94.66 ± 0.44 ^c^	0.272 ± 0.008 ^a^
DE 10	4.81 ± 0.24 ^ab^	95.19 ± 0.24 ^bc^	0.260 ± 0.015 ^a^
DE 19	4.31 ± 0.07 ^bc^	95.69 ± 0.07 ^ab^	0.235 ± 0.003 ^b^

Mean ± standard deviation. Different letters indicate a significant difference (*p* < 0.05).

**Table 4:** Effects of different polysaccharide carriers on pH and titratable acidity of spray-dried coconut yogurt.

Maltodextrin	pH ^ns^	Titratable Acidity ^ns^ (%)
RD	4.05 ± 0.05	1.42 ± 0.09
DE 2	4.06 ± 0.04	1.44 ± 0.10
DE 10	4.06 ± 0.06	1.39 ± 0.07
DE 19	4.04 ± 0.06	1.44 ± 0.06

Mean ± standard deviation. ns = no significant difference (*p* ≥ 0.05).

**Table 5:** Effects of different polysaccharide carriers on organic acids of spray-dried coconut yogurt.

Maltodextrin	Citric Acid (mg/g)	Malic Acid (mg/g)	Lactic Acid (mg/g) ^ns^
RD	not detected	4.94 ± 0.13 ^c^	19.97 ± 1.41
DE 2	0.28 ± 0.02 ^a^	6.77 ± 0.03 ^a^	19.98 ± 1.12
DE 10	0.27 ± 0.04 ^a^	6.83 ± 0.37 ^a^	21.07 ± 0.33
DE 19	0.17 ± 0.01 ^b^	6.10 ± 0.24 ^b^	21.48 ± 0.66

Mean ± standard deviation. Different letters indicate a significant difference (*p* < 0.05). ns = no significant difference (*p* ≥ 0.05).

## Data Availability

The original contributions presented in the study are included in the article, further inquiries can be directed to the corresponding author.
